# Comparison between Carprofen and Meloxicam for Post-Neutering Pain Management in Pet Rabbits

**DOI:** 10.3390/vetsci11060257

**Published:** 2024-06-05

**Authors:** Matteo Serpieri, Chiara Ottino, Giuseppe Bonaffini, Penelope Banchi, Giuseppe Quaranta, Mitzy Mauthe von Degerfeld

**Affiliations:** 1 Centro Animali Non Convenzionali (CANC)—Department of Veterinary Sciences, University of Turin, 10095 Grugliasco, Italy; matteo.serpieri@unito.it (M.S.); chiara.ottino@unito.it (C.O.); giuseppe.bonaffini@unito.it (G.B.); giuseppe.quaranta@unito.it (G.Q.); 2 Department of Veterinary Sciences, University of Turin, 10095 Grugliasco, Italy; penelope.banchi@unito.it; 3 Department of Internal Medicine, Reproduction and Population Medicine, Faculty of Veterinary Medicine, Ghent University, 9820 Merelbeke, Belgium

**Keywords:** analgesia, carprofen, meloxicam, neutering, pain scale, rabbit

## Abstract

**Simple Summary:**

In this study, an investigation was conducted to assess postsurgical pain management in pet rabbits. The efficacy of two pain-relief drugs, carprofen and meloxicam, was compared. Fifty rabbits underwent neutering surgery, and their pain levels were evaluated using a composite scale. The results indicated similar effectiveness of both drugs in alleviating postoperative pain. No adverse effects from either drug were observed. The use of both carprofen and meloxicam can be considered safe in clinical practice, enhancing the welfare of pet rabbits undergoing surgical neutering.

**Abstract:**

Surgical neutering in pet rabbits is common practice to prevent reproduction and associated health issues. Adequate postoperative pain management is crucial for recovery, yet effective methods in clinical settings remain underexplored. This study compared the analgesic effects of carprofen and meloxicam in pet rabbits undergoing surgical neutering. Fifty rabbits of varied demographics were included, with pain assessed using the Centro Animali Non Convenzionali Rabbit Scale (CANCRS). Rabbits were allocated to receive postoperative 2 mg kg^−1^ carprofen or 1 mg kg^−1^ meloxicam by subcutaneous injection. Anesthesia was induced with an intramuscular combination of ketamine (20 mg kg^−1^), medetomidine (0.4 mg kg^−1^), and butorphanol (0.2 mg kg^−1^), and ovariectomy or orchiectomy were performed. The CANCRS scale was used to assess pain by evaluating the rabbit preoperatively, 6 h postoperatively, and at three time points the following day. Times of return to spontaneous feeding and fecal production were also recorded. No statistically significant difference was found between treatment groups based on CANCRS scores and resumption of food intake and fecal output. No clinically detectable adverse effects were noted. While limitations include the use of a single pain assessment scale and the absence of a placebo control group, the results suggest that both carprofen and meloxicam can be viable options in clinical practice. Further research utilizing diverse pain assessment methods is warranted to enhance understanding and optimize pain management strategies for rabbits undergoing surgical procedures.

## 1. Introduction

Surgical neutering is a routine procedure that most pet rabbits undergo to prevent reproduction and onset of unwanted behaviors and hormone-induced diseases, such as uterine adenocarcinoma and mammary neoplasms [1]. After the surgical procedure, adequate postoperative pain management is essential to ensure good recovery and resumption of physiological metabolic and gastrointestinal activities, particularly important features in the clinical management of pet rabbits [2]. Untreated pain can result in activation of the sympathetic nervous system, resulting in negative effects on tissue perfusion, immune response, wound healing, and gastrointestinal motility [3]. A delay in the resumption of food intake and fecal output may cause serious negative metabolic consequences, resulting in rabbit gastrointestinal stasis syndrome, thus increasing postoperative morbidity and mortality [4,5]. Alterations in food intake and fecal output can indeed be considered as indirect markers of the presence of pain in lagomorphs and rodents [2,6].

To adequately treat pain, its presence must be recognized. Recently, composite scales have been developed for the evaluation of pain in rabbits, evaluating facial expressions and physiological and behavioral parameters [7,8,9]. The first composite scale to be developed for rabbits is the Centro Animali Non Convenzionali Rabbit Scale (CANCRS), which is proven to be valid and reliable, and is subsequently adjusted to evaluate the presence of abdominal pain and to identify a cut-off score above which treatment is recommended [10].

In the management of postoperative pain in companion animals, nonsteroidal anti-inflammatory drugs (NSAIDs) have a well-known role, as they produce analgesia resulting from the inhibition of cyclo-oxygenase (COX) [11]. Among these drugs, meloxicam and carprofen are widely used in rabbits in clinical practice, and both are preferential COX-2 inhibitors [3,12]. Despite their common use, these drugs have been studied and evaluated mainly in experimental conditions, often in laboratory animals with standard characteristics [12], while in clinical practice pet rabbits show a wide range of breeds, sizes, and temperaments, which can affect assessments [13]. Furthermore, while the clinical effects of meloxicam and carprofen have been compared and reported in dogs and cats [14,15,16,17], such a comparison has not been performed in pet rabbits.

When dealing with exotic animals in clinical practice, the choice for drugs and dosages is often based on the consultation of formularies, and a specific one (Exotic Animal Formulary) [18] is the most selected [19]. However, reported dosages ranges can be extremely wide, and the references in this formulary often do not cite primary resources [19]. Therefore, the selected dosage for a drug could be quite different among colleagues, and the effects could not always be predictable.

As rabbit neutering is a routine procedure in clinical practice and NSAIDs are commonly employed for postsurgical pain management in this species, an evaluation of the effects of these drugs from a clinical point of view is needed. The aim of the present study was to clinically compare the analgesic effects of carprofen and meloxicam in pet rabbits undergoing surgical neutering.

## 2. Materials and Methods

Approval was given by the Ethical committee of the Department of Veterinary Science of the University of Turin (report N. 247/2022).

The use of a β of 0.80, an α error of 0.05, and a difference in mean CANCRS score between groups equal to two points was considered for sample size calculation. Results indicated that the use of at least six subjects for each group would prevent a type II error. Sample size was calculated using the G*Power software (version 3.1.9.6).

The study was conducted using four groups (group assignment explained at Section 2.3). To prevent data loss due to exclusion of patients, the number of rabbits exceeded the sample size calculation, and the study included 50 client-owned pet rabbits (females *n* = 28, males *n* = 22) of various age, weight, and breeds, undergoing anesthesia for elective surgical neutering (i.e., ovariectomy and orchiectomy). Based on a thorough physical examination, only rabbits classified as American Society of Anesthesiologists’ (ASA) physical status grade I were included, while obese, cachectic, or ill subjects were excluded.

### 2.1. Surgical and Anesthetic Procedures

All surgical procedures were performed in the morning, between 8 and 10 a.m. The rabbits were hospitalized the day before the procedure to reduce the stress associated with transport and to let the rabbit become familiar with the place. During hospitalization, each subject was housed indoors (temperature: about 22 °C) in a stainless-steel single cage typically used for pet animals (Stainless Steel Single Cage, 30″ W × 36″ H, Shor-Line, Kansas City, KS, USA), individually. Newspaper sheets were used as substrate and changed at least once a day during cage cleaning. The rabbits were provided with hay, rabbit pellet food, fresh vegetables, and water (both in a metal bowl and a drinking bottle). The subjects were not fasted before the procedures.

To induce anesthesia, a combination of 20 mg kg^−1^ ketamine (Ketavet 100, MSD Animal Health S.r.l., Segrate, Milan, Italy), 0.4 mg kg^−1^ medetomidine (Medeson, Industria Italiana Integratori Trei S.p.A., Livisto, Rio Saliceto, Reggio-Emilia, Italy), and 0.2 mg kg^−1^ butorphanol (Nargesic^®^ 10 mg/mL, Acme S.r.l., Corte Tegge-Cavriago, Reggio-Emilia, Italy) was intramuscularly administered to all subjects [20]. After the loss of the righting reflex, each rabbit was positioned on a heating pad in dorsal recumbency and connected to a multiparameter monitoring system (Infinity Delta^®^, Dräger Italia S.p.A., Corsico, Italy) to monitor vital parameters via electrocardiography and pulse oximetry. Rabbits received 1.5 L/min of 100% oxygen through a face mask (anesthetic face mask, S, Jørgen Kruuse A/S, Langeskov, Denmark) using a nonrebreathing respiratory system (Bain coaxial breathing system, Intersurgical, Wokingham, UK). In females, ovariectomy was performed via median laparotomy. In males, orchiectomy was performed with scrotal approach and open-closed technique [21]. All surgical and anesthesiologic procedures were performed by the same operators (G.B. and M.M.v.D., respectively).

At the end of the procedure, 2 mg kg^−1^ atipamezole (Sedastop^®^ 5 mg/mL, Ecuphar Italia S.r.l., Milan, Italy) was intramuscularly administered. Each animal was placed in a cage prepared with soft blankets to avoid trauma during recovery. An infrared heating lamp (InfraRed Industrial Heat Incandescent, Philips Lighting, Signify Italia S.p.A., Milan, Italy) was attached to each cage and maintained for 30 min. The animals were monitored until resumption of righting reflex. The procedural time was calculated from the administration of the anesthetic mixture to the resumption of righting reflex. Hay, rabbit pellet food, fresh vegetables, and water (both in a metal bowl and in a drinking bottle) were given to each animal after righting reflex was regained. Thereafter, the cage was cleaned and prepared in the same manner as for the preoperative period. The discharge of the rabbits was scheduled for the third day postsurgery, to conduct evaluations during the first and second days (see Section 2.3).

### 2.2. Administration of NSAIDs and Other Drugs

Immediately before the administration of atipamezole, and at the end of the surgical procedure, all rabbits were administered an NSAID subcutaneously (SC) for pain management by the anesthetist. The rabbits were randomly allocated into two groups for each sex (www.randomizer.org; accessed on 10 November 2022). A dose of 2 mg kg^−1^ carprofen (Rimadyl^®^ Iniettabile 50 mg/mL; Zoetis Italia S.r.l., Rome, Italy) was administered to the rabbits sorted into groups COR (11/22 male subjects undergoing orchiectomy) and COV (14/28 female subjects undergoing ovariectomy), whereas 1 mg kg^−1^ meloxicam (Meloxidyl^®^ 5 mg/mL, Ceva Salute Animale S.p.A., Milan, Italy) was administered to rabbits belonging to groups MOR (11/22 male subjects undergoing orchiectomy) and MOV (14/28 female subjects undergoing ovariectomy). Doses for the NSAIDs were chosen based on ranges found in the Exotic Animal Formulary [22] and previous experience of the Authors.

All rabbits were then subcutaneously administered 5 mg kg^−1^ enrofloxacin (Enrovet, Industria Italiana Integratori Trei S.p.A., Livisto, Rio Saliceto, Reggio-Emilia, Italy) once a day for 5 days.

In case of anorexia, following resumption of righting reflex, each rabbit received assisted feeding via syringe (15 mL/kg, Critical Care Herbivore, Oxbow Animal Health, Omaha, NE, USA) three times a day until spontaneous feeding resumed.

### 2.3. Pre- and Postoperative Evaluations and Postoperative Rescue Analgesia

In the postoperative period, pain scores were longitudinally determined and recorded together with the times of return to spontaneous feeding and fecal production.

The CANCRS composite scale (range: 0 to 24) was used to assess the presence of pain [7]. This scale is based on the integration of the Rabbit Grimace Scale [23] with the evaluation of clinical and physiological parameters, specifically designed for the assessment in a clinical environment. The evaluated parameters are reported in Table A1, and the specific form to be fulfilled can be found in the Supplementary Materials (File S1).

The evaluations were performed before the surgical procedure (T0), 6 h after the surgical procedure (T1), and the following day at three time points: at 9.00 a.m. (T2) immediately before the NSAID administration, at 1.00 p.m. (T3), and at 6.00 p.m. (T4). The CANCRS scale evaluations were always performed by the same operator (M.S.), blinded to the allocated group.

For each rabbit, times of return to spontaneous feeding and fecal production were recorded. Time was assessed in hours from atipamezole administration by visually inspecting each rabbit and its cage every hour, for the presence of fecal pellets (at least 10 pellets), or checking if the rabbit was eating (visible chewing) or had eaten the food at its disposal. These times were assessed by veterinary medicine students, blinded to the allocated group.

A score equal to 10 obtained using the CANCRS was chosen as cut-off value beyond which postoperative rescue analgesia was required. In that case, the intramuscular administration of 0.2 mg kg^−1^ butorphanol was planned.

### 2.4. Statistical Analysis

Microsoft Excel (Microsoft 365, 2024, Microsoft Corp., Washington, DC, USA) and R (version 4.2.2, R Foundation for Statistical Computing, Vienna, Austria) were used for data management and statistical analysis. Continuous variables did not have normal distribution (Shapiro–Wilk test with *p* < 0.05).

A two-tailed Wilcoxon rank sum test was performed to evaluate homogeneity between groups (COR versus MOR, COV versus MOV) for the following variables: weight, age, and procedural time.

A two-tailed Wilcoxon rank sum test was performed to compare time of return to spontaneous feeding, time to return to fecal production, and CANCRS scores at each time point between the groups (COR versus MOR, COV versus MOV).

Statistical significance was set at *p* < 0.05.

## 3. Results

Variables are reported as median and interquartile range. The results regarding age, weight, procedural time, and times of return to spontaneous feeding and fecal production in rabbits undergoing orchiectomy (COR, MOR) are reported in Table 1, while the results of the same parameters in rabbits undergoing ovariectomy (COV, MOV) are reported in Table 2. No statistical differences were found significant for any parameters between the groups.

The results regarding the CANCRS scores in rabbits undergoing orchiectomy (COR, MOR) at different time points are reported in Table 3, while the results of the same parameters in rabbits undergoing ovariectomy (COV, MOV) are reported in Table 4. No statistically significant differences were found for any clinical parameter between the groups. Results regarding the specific parameters evaluated using CANCRS can be found in the Supplementary Materials (Table S1).

No rabbits were excluded from the study, and all rabbits were discharged as planned with no complications.

## 4. Discussion

No significant differences were found for comparisons between treatment groups, suggesting that the analgesic effect of postoperative meloxicam and carprofen was equivalent in rabbits undergoing surgical neutering. These results are in accordance with results obtained in other studies involving dogs and cats [14,15,16,17]. However, in the studies by Slingsby and Waterman-Pearson [14,15], cats treated with meloxicam required rescue analgesia more frequently compared to cats treated with carprofen after ovariohysterectomy, while the opposite occurred in the study by Hernandez-Avalos et al. [24] in dogs undergoing the same procedure. These differences and variability may be linked to species-specific characteristics in drugs metabolism or to the use of suboptimal therapeutic doses. In this study, a cut-off score of 10 was chosen a priori to resort to postoperative rescue analgesia. This was set considering the cut-off score of 7 that had been previously identified for treating animals with abdominal pain, but not associated with surgical procedures [10]. It was assumed that, especially in the first postoperative hours, the scores could have been higher than this cut-off and, to avoid interfering with the assessments, the score of 10 was considered more adequate in case of pain related to a surgical procedure. Nevertheless, as reported in Table 3 and Table 4, the maximum scores obtained were lower than 10 and the overall median scores were lower than 7; therefore, it was not necessary to further administrate analgesic drugs.

The scores at different time points showed no differences between the groups; therefore, the effects of meloxicam and carprofen are assumed to be equivalent at the chosen doses. In female rabbits undergoing ovariectomy, a median score equal to 4 was obtained at T1, with lesser median scores at the other evaluated times, in both groups COV and MOV. It is possible that the analgesic effects of the used NSAIDs became evident a few hours after the administration of the drugs, thus potentially creating a period in which the analgesic effects of butorphanol and ketamine had already diminished, and those of carprofen or meloxicam were not yet fully present. If so, the peak in pain scores at T1 may be avoided by preoperative administration of the NSAIDs. Furthermore, it has been suggested that atipamezole can reduce the postoperative analgesic activity of butorphanol in rats [25,26]. These assumptions have not been verified in rabbits, but it is possible that the use of atipamezole reduced the analgesic effects of butorphanol, as well as those of medetomidine, exacerbating postoperative pain before the onset of effects of the NSAIDs.

In addition to relying on pain assessment tools, research involving herbivorous mammals frequently considers the restoration of regular feeding patterns and fecal output as crucial indicators for assessing the efficacy of analgesic treatments, as pain presence can lead to a slowdown in normal gastrointestinal function [2,12,27,28]. This assessment is typically taken into account in studies concerning perioperative pain management in rabbits [2,12,27,28,29]. In this study, all animals showed a return to spontaneous feeding and fecal production within the day. The median resumption times were higher in rabbits undergoing orchiectomy treated with carprofen compared to those treated with meloxicam, while the opposite occurred in rabbits undergoing ovariectomy (Table 1 and Table 2), although this difference is not to be considered significant. In fact, the statistical analysis has only been performed for comparisons between groups for the same type of surgery (COV vs. MOV, COR vs. MOR), given that the surgical procedures were different. However, some considerations can be made. To the authors’ knowledge, no studies that evaluate differences in the effectiveness of drugs according to gender in rabbits are present in the literature, although some sex-related differences in the metabolism and effects of NSAIDs and other drugs have been reported in human and veterinary medicine [30,31,32,33]. As male rabbits have open inguinal rings throughout life and testes may be retracted in the abdomen, it could be assumed that traction of ovaries or testicles during ovariectomy and orchiectomy would produce similar visceral pain in rabbits [34,35]. On the other hand, some researchers hypothesized that the pain following ovariectomy is greater than that following orchiectomy [14], and it is possible that meloxicam provides less analgesia than carprofen in case of abdominal surgery in rabbits. However, the observed differences may be accidental and related to a low sample size.

In addition, it is noteworthy that the chosen anesthesiological protocol may impact the restoration of normal gastrointestinal functionality. It has been reported that a protocol based on ketamine and medetomidine prior to anesthesia induction with isoflurane is more likely to induce a reduction in gastric and intestinal peristalsis, compared to the use of ketamine and midazolam [36]. In the present study, in addition to ketamine and medetomidine, butorphanol was utilized as part of the anesthesiological protocol. This molecule is commonly deemed safe when applied in rabbit anesthesia, with few adverse effects [13,20]. One study reported a temporary reduction in gastric motility following the administration of 10 mg kg^−1^ of butorphanol intramuscularly [37]. However, this dose is 20 times higher than that used in this study (0.2 mg kg^−1^); hence, the results may not be directly comparable to those of the cited study. Nevertheless, the same anesthesiological protocol was employed in all subjects in this study to avoid group differences related to this procedure.

In the present study, no clinically detectable short-term adverse effects related to the use of the two NSAIDs were noted. Although adverse effects of meloxicam and carprofen are frequently reported in other species, the occurrence in rabbits is rare, particularly if appropriate doses are used [3]. The pharmacokinetics of carprofen administered at 2 mg kg^−1^ SQ, as in this study, has been evaluated in a sample of New Zealand White rabbits, but the safety of this dose has not been assessed [38]. Carprofen administered at 5 mg kg^−1^ SQ did not produce significant adverse effects on food and water intake and on fecal and urinary output [39]. On the other hand, no obvious adverse effects have been demonstrated after administration of 1 mg kg^−1^ meloxicam in rabbits in several studies [40,41,42,43]. This is widely reported in the literature, with studies demonstrating the drug’s safety even at a dose of 1.5 mg kg^−1^ for 5 consecutive days administered orally [12,44]. The doses of meloxicam reported in the literature are quite broad, but for efficacy in rabbits, doses ranging from 0.6 to 1 mg kg^−1^ are suggested, as in this case [12,29]. The doses used, therefore, are unlikely to cause adverse effects; however, further studies could verify the possible onset of subclinical alterations that were not considered in the cited studies.

As both drugs produced similar effects, the choice of the NSAID to be used could be based on several factors. In veterinary clinical practice, the familiarity with the use of one drug over another often plays an important role and could influence the choice [45,46]. Moreover, the overall volume of drug could also guide the choice: with the pharmaceutical formulations used in this study, the volume of carprofen to be administered was 0.04 mL kg^−1^, while the volume of meloxicam was 0.2 mL kg^−1^. This difference is of little relevance in case of subcutaneous administration, but a smaller volume may be preferable in case of intramuscular administration (one of the possible routes found in Exotic Animal Formulary for both carprofen and meloxicam [22]), to reduce iatrogenic tissue damage and discomfort related to the injection in an awake subject [47,48]. The final volume can also raise the cost of the drug, an important factor in veterinary clinical practice [49]: with the commercial pharmacological formulations and doses used at the time of the present study, the cost per rabbit was lower with the use of carprofen compared to meloxicam. Nonetheless, considering that postoperative anti-inflammatory therapy often needs to be continued at home by owners, the availability of drugs that are easily administrable by nonveterinary personnel plays a crucial role [3]. At present, no oral suspension of carprofen is commercially available in Italy, but the drug is, instead, marketed in the form of tablets. On the contrary, the availability of oral suspensions of meloxicam is wide. Administration of tablets in rabbits is difficult, and suspensions are preferred [50]: for this reason, meloxicam is one of the most prescribed drugs to rabbit owners [12].

The decision to conduct evaluations using only the CANCRS scale may pose a limitation to the present study, since in recent years alternative scales have been developed for pain assessment in rabbits, including the Bristol Rabbit Pain Scale (BRPS) [51] and the Rabbit Pain Behavior Scale (BPRS) [9]. Although the CANCRS demonstrated reliability [10], scoring is based on an assessment of physiological and behavioral parameters and involves, at least in part, direct observation of the patient. This situation can introduce biases in the assessment due to the stress induced by the presence of an observer, and rabbits may not exhibit behavior related to painful condition in an unfamiliar environment [2,52]. An advantage of the BRPS lies in the lack of need for collecting physiological data that involve direct contact with the animal (i.e., heart rate, reaction to palpation of the painful area), thus avoiding causing stress related to manipulation [51]. However, the BRPS was also designed for direct observation of rabbits, although video recordings can also be used [51]. The BPRS scale, on the other hand, was developed and validated for New Zealand White rabbits, and therefore may not necessarily be applicable in a clinical setting with pet animals [9]. Moreover, while comparisons with other scales based on different methodologies could yield interesting results, the CANCRS scale was chosen in the phase of development of the study, being the first developed, and, thus, was used throughout the data collection period. 

As mentioned, there is a possibility that subjects may not exhibit their normal behavioral patterns in an unfamiliar environment [2,52]. However, scales such as the CANCRS and the BRPS have been specifically validated to work in such contexts, considering the need for pain assessment in rabbits that must be hospitalized at a veterinary clinic [10,51]. Nevertheless, to further address this issue during a comparative evaluation, it was chosen in this case to use the same cages and setup for all subjects, so as not to have iatrogenic modifications related to different housings.

The decision to use a composite scale, instead of the evaluation of a biomarker such and serum cortisol or glucose, was based on the fact that even anesthesia and handling can cause transient increases in both glucose and cortisol concentrations [53,54]. Even the simple manipulation required to obtain samples for biomarker analysis could cause alterations in their concentration, also depending on the character and temperament of the individual subject. In any case, the combined evaluation of specific markers and different composite pain scales can be the subject of future studies for a more objective assessment of painful conditions in rabbits.

Another possible limitation is the lack of a control placebo group to assess differences between treated and untreated animals. This was due to ethical reasons: as mentioned, pain can lead to anorexia and start a vicious circle of gastroenteric stasis that is particularly dangerous for rabbits, due to their physiology [4]. This makes the risks associated with NSAIDs use lower than those arising from the lack of any analgesia. 

## 5. Conclusions

Carprofen and meloxicam produced similar effects in postoperative pain management in rabbits undergoing elective surgical sterilization in clinical practice. The absence of obvious clinical adverse effects makes these drugs a possible choice. Further work should involve the use of different evaluation methods for postoperative pain assessment in rabbits.

## Figures and Tables

**Table 1:** Differences in weight, age, procedural time, and times of return to spontaneous feeding and fecal production in rabbits undergoing orchiectomy treated either with subcutaneous carprofen (COR) or meloxicam (MOR) postsurgical administration. Results are reported as median (interquartile range, IQR).

Variable	Group COR	Group MOR	*p* Value
Weight (kg)	1.50 (1.30–1.95)	1.80 (1.35–1.90)	0.869
Age (months)	7 (6–8)	12 (12–27)	0.069
Procedural time (min)	35 (32–52)	49 (40–63)	0.167
Return to spontaneous feeding (h)	6 (5–8)	4 (3–6)	0.103
Return to fecal production (h)	8 (6–9)	4 (3–7)	0.138

**Table 2:** Differences in weight, age, procedural time, and times of return to spontaneous feeding and fecal production in rabbits undergoing ovariectomy treated either with subcutaneous carprofen (COV) or meloxicam (MOV) postsurgical administration. Results are reported as median (interquartile range, IQR).

Variable	Group COV	Group MOV	*p* Value
Weight (kg)	1.65 (1.53–1.90)	1.70 (1.45–1.92)	0.982
Age (months)	7 (7–9)	7 (7–9)	0.889
Procedural time (min)	53 (49–60)	50 (46–60)	0.597
Return to spontaneous feeding (h)	6 (4–10)	9 (6–15)	0.087
Return to fecal production (h)	6 (6–12)	9 (7–13)	0.173

**Table 3:** Comparison of CANCRS scores between groups in rabbits undergoing orchiectomy (carprofen = COR and meloxicam = MOR). Scores are reported as median (interquartile range, IQR). Minimum and maximum values are also reported. The evaluations were performed before the surgical procedure (T0), 6 h after the surgical procedure (T1), and the following day at three time points: at 9.00 a.m. (T2) immediately before the NSAID administration, at 1.00 p.m. (T3), and at 6.00 p.m. (T4).

	Group COR		Group MOR		
Score (Min–Max)	Median (IQR)	Min–Max	Median (IQR)	Min–Max	*p* Value
T0 (0–24)	3 (2–3)	1–4	3 (2–3)	0–4	0.972
T1 (0–24)	3 (3–5)	1–6	2 (2–2)	1–6	0.343
T2 (0–24)	2 (1–3)	0–5	2 (2–3)	1–4	0.709
T3 (0–24)	3 (1–3)	0–5	2 (1–3)	0–3	0.453
T4 (0–24)	3 (1–3)	0–3	2 (1–2)	0–3	0.495

**Table 4:** Comparison of CANCRS scores between treatment groups in rabbits undergoing ovariectomy (carprofen = COV and meloxicam = MOV). Scores are reported as median (interquartile range, IQR). Minimum and maximum values are also reported. The evaluations were performed before the surgical procedure (T0), 6 h after the surgical procedure (T1), and the following day at three time points: at 9.00 a.m. (T2) immediately before the NSAID administration, at 1.00 p.m. (T3), and at 6.00 p.m. (T4).

	Group COV		Group MOV		
Score (Min–Max)	Median (IQR)	Min–Max	Median (IQR)	Min–Max	*p* Value
T0 (0–24)	3 (2–3)	0–4	3 (2–3)	0–4	0.884
T1 (0–24)	4 (2–5)	0–8	4 (2–5)	0–9	0.780
T2 (0–24)	2 (1–3)	0–3	3 (2–4)	0–6	0.235
T3 (0–24)	2 (1–3)	0–4	2 (1–3)	0–4	0.720
T4 (0–24)	1 (0–2)	0–7	2 (0–2)	0–4	0.567

## Data Availability

All data are contained within the Article.

## Supplementary Materials

The following supporting information can be downloaded at: https://www.mdpi.com/article/10.3390/vetsci11060257/s1, File S1: CANCRS form to be fulfilled for pain assessment for each rabbit; Table S1: Data for each parameter evaluated for CANCRS for rabbits undergoing ovariectomy or orchiectomy.

## Appendix A

**Table A1:** CANCRS composite rabbit pain scale [2,7].

Category	Parameters to Score	Score Range
Rabbit Grimace Scale	Orbital tightening	0–2
	Cheek flattening	0–2
	Nostril shape	0–2
	Whisker shape and position	0–2
	Ear shape and position	0–2
Clinical Parameters	Pupil dilation	0–1
	Heart rate % increase (based on 250 bpm)	0–2
	Respiratory rate (based on 60 breaths per min)	0–2
	Respiratory pattern	0–1
	Palpation of the painful area	0–2
	Mental status	0–2
	Vocalizations	0-3
