# Supplementary material for: Comparison between Carprofen and Meloxicam for Post-Neutering Pain Management in Pet Rabbits
Source: Vet Sci. 2024 Jun 5;11(6):257. doi: 10.3390/vetsci11060257 (PMC11209068; doi:10.3390/vetsci11060257)
Supplement: Supplementary file 1 [file vetsci-11-00257-s001.zip › File S1.pdf]

Scheda CANCRS

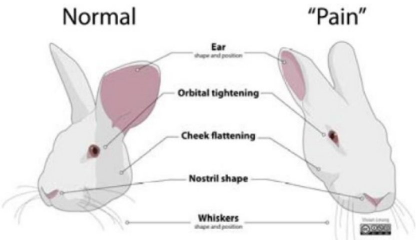

Nome Coniglio	Proprietario	C.C.	Età	Sesso	Razza

Giorno	h inizio anest	h fine chirurgia	H risveglio	Peso

Tipo Chirurgia	Tipo Analgesia	gg/h Ripresa Appetito	gg/h Ripresa defecazione

Altri Farmaci	Dose	Via

		Pre-chirurgia	6h Post	Giorno 2, h9	Giorno 2, h13	Giorno 2, h18
**Orbital tightening**	Not present	0	0	0	0	0
	Moderately present	1	1	1	1	1
	Obviously present	2	2	2	2	2
**Cheek flattening**	Not present	0	0	0	0	0
	Moderately present	1	1	1	1	1
	Obviously present	2	2	2	2	2
**Nostril shape**	Not present	0	0	0	0	0
	Moderately present	1	1	1	1	1
	Obviously present	2	2	2	2	2
**Whisker position**	Not present	0	0	0	0	0
	Moderately present	1	1	1	1	1
	Obviously present	2	2	2	2	2
**Ear position (no per Ariete)**	Not present	0	0	0	0	0
	Moderately present	1	1	1	1	1
	Obviously present	2	2	2	2	2
**Pupil dilation**	Not present	0	0	0	0	0
	Moderately present	1	1	1	1	1
	Obviously present	2	2	2	2	2
**Heart rate**	Increases ≤20% of the physiological value (250 bpm): <300 bpm	0	0	0	0	0
	Increases between 20% and 50% of the physiological value (250 bpm): 300-375 bpm	1	1	1	1	1
	Increases > 50% of the physiological value (250 bpm): >375 bpm	2	2	2	2	2
**Respiratory rate**	≤ 60 bpm	0	0	0	0	0
	61 - 72 bpm	1	1	1	1	1
	73 - 90 bpm	2	2	2	2	2
	> 90 bpm	3	3	3	3	3
**Respiratory pattern**	Eupneic	0	0	0	0	0
	Dyspneic	1	1	1	1	1
**Palpation of the painful area**	No reaction	0	0	0	0	0
	Reaction during the palpation	1	1	1	1	1
	Reaction before the palpation	2	2	2	2	2
**Vocalization**	Absent	0	0	0	0	0
	When touched	1	1	1	1	1
	Intermittent vocalization without any contact	2	2	2	2	2
	Continuous vocalization without any contact	3	3	3	3	3
**Mental status**	Normal	0	0	0	0	0
	Depression	1	1	1	1	1
	Obtundation	2	2	2	2	2
**Totale**						
